# Each niche has an actor: multiple stem cell niches in the preterm kidney

**DOI:** 10.1186/s13052-015-0187-6

**Published:** 2015-10-15

**Authors:** D. Fanni, A. Sanna, C. Gerosa, M. Puddu, G. Faa, V. Fanos

**Affiliations:** Department of Pathology, University of Cagliari, via Ospedale 56, 09100 Cagliari, Italy; Department of Surgery, Neonatal Intensive Care Unit, Puericulture Institute and Neonatal Section, Policlinico Monserrato, Azienda Ospedaliera Universitaria di Cagliari, University of Cagliari, 09042 Monserrato, Italy

**Keywords:** Neonatal human kidney, Preterm human kidney, Fetal human kidney, Stem cell niches, Regenerative medicine

## Abstract

The preterm kidney cannot be simply considered as a kidney small in size: as compared to the adult kidney, the developing organ of the preterm infant is characterized by marked differences regarding the architecture and cell components. At macroscopy, fine linear demarcations indenting the renal surface characterize the fetal and preterm kidney. At microscopy, multiple major architectural changes differentiate the developing kidney from the adult one: a large capsule with a high cellularity; the branching ureteric bud, extending from the hilum towards the renal capsule; striking morphological differences among superficial (just born) and deep (more mature) glomeruli; persistence of remnants of the metanephric mesenchyme in the hylum; incomplete differentiation of developing proximal and distal tubules. At cellular level, kidneys of preterm infants are characterized by huge amounts of stem/precursor cells showing different degrees of differentiation, admixed with mature cell types. The most striking difference between the preterm and adult kidney is represented by the abundance of stem/progenitor cells in the former. Multiple stem cell niches may be identified in the preterm kidney, including the capsule, the sub-capsular nephrogenic zone, the cap mesenchyme embracing the ureteric bud tips, the cortical and medullary interstitium, and the hilar zone in proximity of the ureteric origin. The sub-capsular area represents the major stem cell niche in the prenatal kidney. It has been defined “blue strip”, due to the scarcity of cytoplasm of the undifferentiated stem/progenitors, which appear as small cells arranged in a solid pattern. All these data taken together, the morphological approach to the analysis of the preterm kidney appears completely different from that typically utilized in kidney biopsies from adult subjects. Such a different structure should be taken into account when evaluating renal function in a preterm infant in clinical practice. Moreover, a better knowledge of molecular biology of the blue strip stem/progenitor cells could be at the basis of a new “endogenous” regenerative medicine, finalized to maintain and protect the nephrogenic potential of preterm infants till the 36th week of post-conceptional age, allowing them to escape oligonephronia and chronic kidney disease later in life.

## Introduction

The development of the human kidney is a process which originates from three embryonic excretory organs, the pronephros, the metanephros, and the metanephros [1]. The definitive human kidney develops from the metanephric mesenchyme, the first mesenchymal component of the urogenital system that, through a process of mesenchymal-epithelial transition, gives rise to all the epithelial components of the proximal nephron [2]. The metanephros takes origin from two components: *i*) the ureteric bud (UB), a branching epithelial tube originating from the Wolffian duct, and *ii*) the metanephric mesenchyme, which originates from the intermediate mesenchyme [3]. In the metanephric mesenchyme reside self-renewing stem cells that are induced to form all cell types of the nephron [4]. While epithelial cords originating from the UB are branching into the metanephric mesenchyme, some metanephric mesenchymal cells, including self-renewing progenitors [5], condensate and aggregate around the tips of the epithelial branches, differentiating into the cap mesenchymal cells [6, 7]. Cap mesenchyme progressively undergoes mesenchymal-to epithelial transition [8, 9], giving rise to most of the epithelia of the proximal nephron [10]. The cap mesenchyme develops into the renal vesicle, the first mesenchyme-derived epithelial structure. The renal vesicle gives rise to the comma body, which originates the S-shaped body, from which the glomerulus, proximal and distal tubules, and Henle loops take origin. The distal tubule eventually fuses with the collecting ducts, the only epithelial structure of the mature kidney which originates from the ureteric bud. The region of the ureteric bud external to the metanephric mesenchyme gives rise to the ureter [3].

In recent years, a strong link is emerging between intrauterine development and kidney disease occurring in adulthood, thus reinforcing the theory of a developmental origin of adult health and disease, including nephropaties [11]. Considering kidney development and its relation to adult renal disease, it has been hypothesized that an insult taking place in a specific time window during development may cause a permanent alteration in kidney architecture and function, affecting nephron number, glomerular volume, tubular cell function, vascular permeability, interstitial cell function and so on [12]. In humans, nephrogenesis is complete by week 34–36 of gestation. As a consequence, at term babies normally do not show signs of active nephrogenesis. In preterm newborns, nephrogenesis has been shown to continue for some time after birth, ending within about 4–6 weeks of postnatal life. Our ability to restore low nephron number by compensatory nephrogenesis after birth is actually very limited. As a consequence, preterm infants often show a low nephron number for the rest of their life, as compared to at term newborns [13]. An incomplete nephrogenesis at birth in preterms may be a likely risk factor for developing hypertension and chronic kidney disease (CKD) later in life [14]. The aim of this study was to characterize the macroscopic and microscopic peculiar features of the human preterm kidney as compared to the adult one. In particular the multiple pools of stem/progenitor cells which characterize the preterm kidney will be analyzed in this work.

### Stem cell niches in the preterm human kidney

In recent years, the attention of many research groups as been focused on the presence of a huge amount of stem/progenitor cells in the fetal and preterm kidney [15]. The capsular and the sub-capsular areas in which glomerulogenesis occurs have been first identify as the unique homing for renal stem cells [16]. Multiple molecular factors, including Mouse double minute 2(Mdm2), have been identified able of maintaining the functionality of the nephrogenic niches [17]. In recent studies other niches have been hypothesized, including the Bowman capsule in which Cluster Differentiation of 44 (CD44)-reactive progenitors might exist intermingled with parietal epithelial cells [18], the cortical and medullary interstitium [19], the renal papilla [20] and the hilar regions in proximity of the ureteric emergence [21]. Recently, Mdm2, an ubiquitin ligase of protein 53 (p53), has been demonstrated to play a fundamental role in nephrogenesis, being indispensable for tha maintenance of all the nephrogenic stem cell niches [17].

**Fig. 1:**
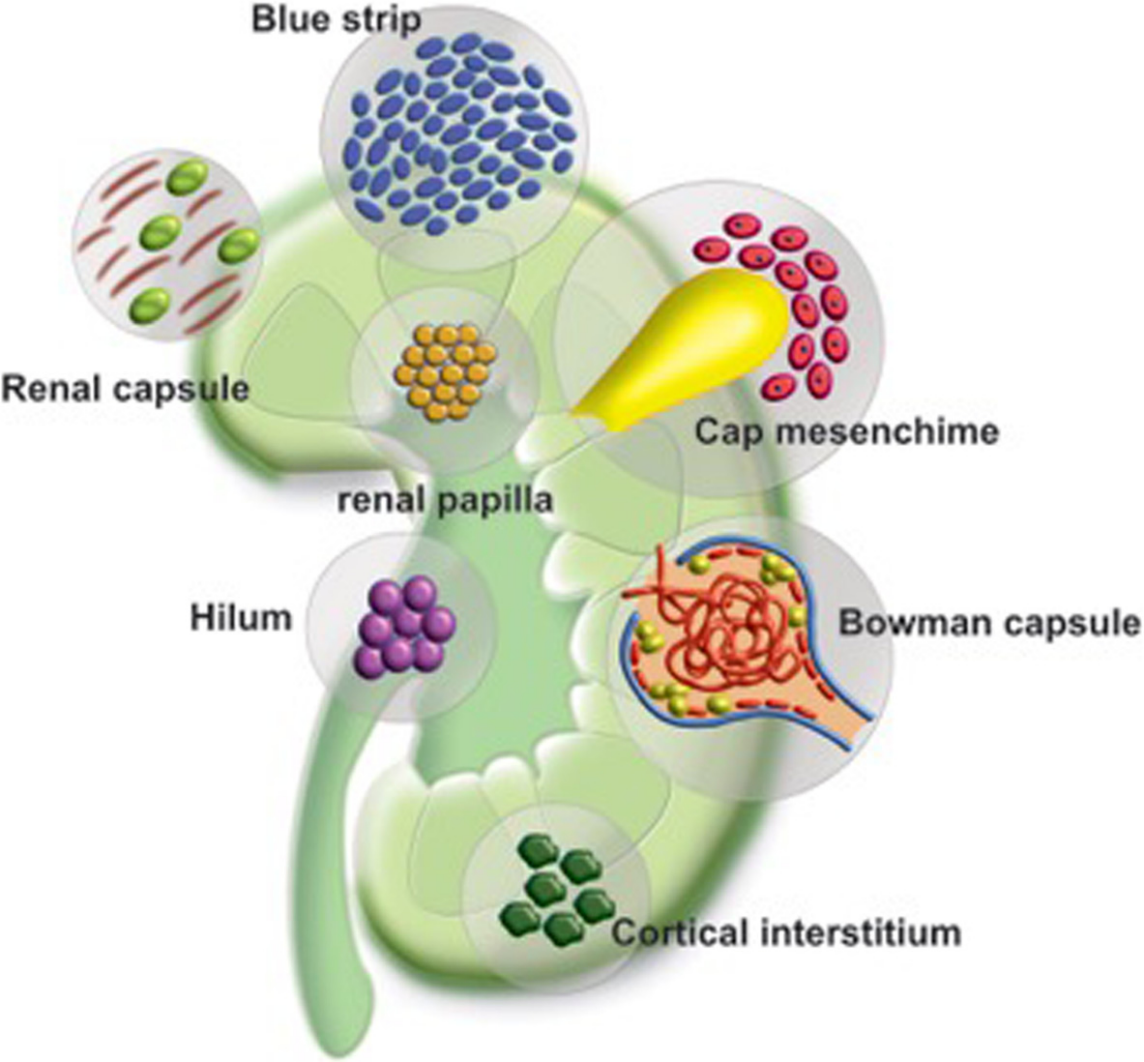
Schematic representation of the putative stem/progenitor cell niches in the neonatal human kidney

Here, the most important findings of all these putative renal stem cells niches will be summarized (Fig. 1).

1. *Renal capsule*. In adult kidney, the renal capsule is considered as a fibrous envelope surrounding the outer surface of the renal parenchyma and covered by a thick layer of adipose tissue. The morphological structure of the renal capsule in the preterm kidney is completely different. It is characterized by a width, by an irregular surface and by a loose appearance. Moreover, the immature renal capsule is characterized by an high number of immature cells admixed with small elongate histiocytes-likes cells (Fig. 2a-b). Capsular stem progenitor cells showed large nuclei, irregular shape with frequent nuclear grows. The amount of stem/progenitor cells inside the preterm renal capsule may change significantly from one case to the next. In some immature kidney they may be arranged in multiple layers whereas in other kidneys the numbers of capsular stem/progenitors appears significantly lower. According with our preliminary studies, the amount of capsular stem/progenitor does not seem to be exclusively related to gestation age. A marked interindividual variability seems to exist among newborns of the same gestational age, suggesting the existence of epigenetic factors able to modulate the extension of intra-capsular stem cell niches. In a recent paper from our group [22], thymosin beta-4 (Tβ4)—an ubiquitous peptide involved in many critical biological activity including cell migration, development and repair—showed a strong immunostaining characteristically localized in the cells of the renal capsule. These preliminary data suggest a role for beta-thymosins, and in particular for Tβ4, in the maintenance of capsular stem cell niches during fetal life and in the preterm baby, indicating this peptide as one of the multiple modulators of nephrogenesis in humans.

**Fig. 2:**
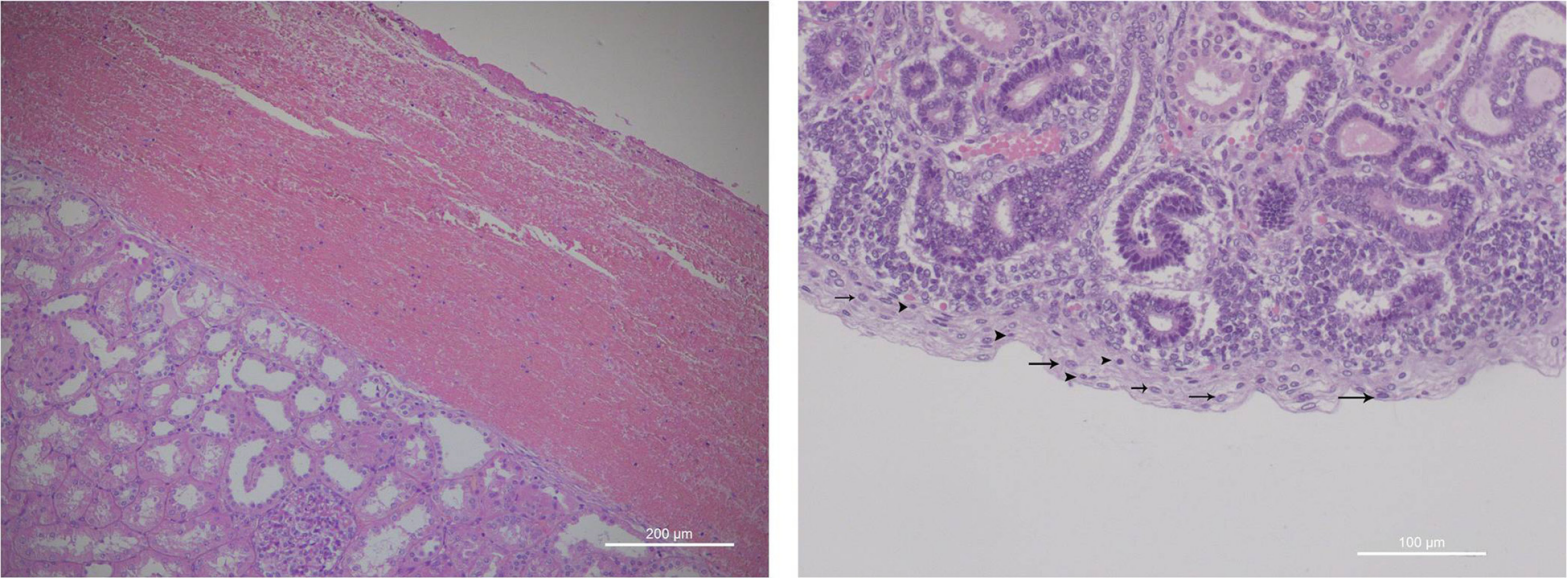
**a** Renal capsule of an adult kidney; **b** renal capsule of a fetal kidney (16 weeks of gestation) containing multiple stem/progenitor cells with large nuclei (*arrows*) and frequent nuclear grows (*arrowheads*)
2. *The blue strip*. In the newborn kidney, the stem/progenitor cells appear as small cells, with a roundish or elongated nucleus and a scant cytoplasm. The scarsity of the cytoplasm is at basis, in hematoxylin and eosin (H&E)-stained sections, of the “blue strip” located under the renal capsule. The blue strip represents the nephron progenitor renal cells of the preterm kidney [23]. These multipotent metanephric mesenchymal cells represent the nephron progenitor population in the human developing kidney, capable to give rise to all segments of new nephrons, except the collecting tubules [3]. The width of the blue strip has been recently suggested to represent the residual nephrogenic potential of each neonatal kidney [24]. The absence of the blue strip in a preterm newborn might indicate the early cessation of nephrogenesis and that no potential nephrogenesis could go on in that kidney. Recently, our group demonstrated that the cells of the blue strip show a peculiar phenotype characterized by immunoreactivity for Mdm2 and Wingless-type MMTV integration site family, member 1 (Wnt1) [25]. A study on a baboon model of preterm birth, perinatal exposure to ibuprofen lead to a significantly reduced nephrogenic zone, evidenced at histology by the reduction of the blue strip width, associated with a early cessation of glomerulogenesis [26].
3. *The cap mesenchyme*. The cap mesenchyme is peculiar pool of stem/progenitor cells, that originate from the metanephric mesenchyme of the subcapsular zone, following induction by the epithelial cells of the ureteric bud tips. At histology, it is possible to observe small groups of undeifferentiated mesenchymal cells condensing around the UB tips, progressively loosing their mesenchymal features and acquiring more strict intercellular relationships. At immunohistochemistry, cap mesenchymal cells are characterized by the acquirance of a strong nuclear immunoreactivity for B-cell lymphoma 2 (BCl-2), an anti-apoptotic protein, whose expression is normally down-regulated in stem/progenitor cells of the blue strip (Fig. 3). The process of differentiation from metanephric mesenchyme toward cap mesenchyme has been recently analyzed at ultra-structural level, revealing the morphological events that take place during the early stages of cap mesenchymal formation [27]. Renal progenitor cells present in the cap mesenchymal nodules exhibited a scanty cytoplasm containing few mitochondria, and a large nucleus with prominent nucleolus. Cap aggregates often showed high variability regarding shape and morphology of their cells: roundish cells were observed in the center of the cap aggregates, whereas thin curved cells were detected at the periphery, twisting around a central cluster and resembling a pine-cone morphology [28]. The initial phases of mesenchymal-epithelial transition (MET) of cap mesenchymal cells have been shown to be marked by the appearance of immunostaining for mucin 1(MUC-1) in the central regions of the cap mesenchymal aggregates paralleling the hypothesis that Bcl-2 overexpression might represent a protective factor for the pluripotent cap cells during the complex process of MET, MUC expression in cap cells might represent a survival response, given the ability of MUC-1 to activate Forkhead Box 3 transcription factor(FOXO3), able to protect cells from oxidative stress [29].

**Fig. 3:**
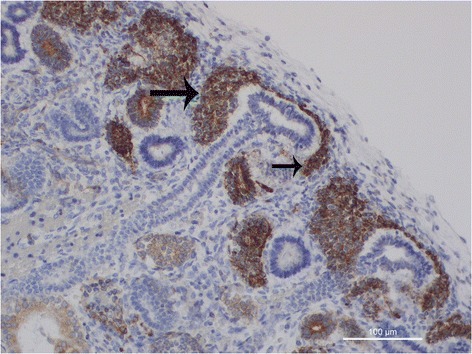
Bcl2 is mainly expressed in the cap-mesenchyme (*arrows*) in a fetal human kidney (16 weeks of gestation)
4. *Cortical interstitium.* A subset of interstitial renal cells have been hypothesized to may represent an important source of renal stem cells oriented toward the epithelial transition [30]. Interstitial multipotent mesenchymal cells have been shown to generate new tubules in adult fish following partial nephrectomy [31]. The morphological study of the preterm kidney cortex might confirm this hypothesis. During development, the renal cortex shows an abundance of intertubular cells, giving rise to a picture completely different from that considered typical of the mature kidney. Whereas in the adult kidney interstitial cells are rare and inconspicuous, in the preterm kidney large cells are easily detectable, with large irregular nuclei. The role of these immature interstitial cells in the development of the human kidney have not been investigated yet. Their similarities, at morphology, with the undifferentiated cells frequently detected in the renal capsule of preterm infants, induce to hypothesize that the interstitium represents a stem cell niche during fetal development. Alternatively, from other groups it has been proposed that a sub-population of surviving intratubular cells might be characterized by multi-potentiality, persisting in the adult kidney and selectively proliferating after tubular damage [19]. According with this suggestion, we may hypothesize that the tubular wall might represent a previously undescribed stem cell niche even in the neonatal kidney. Further studies are needed, at immunohistochemical level, in order to verify if tubular cells show some inter-individual variability regarding immunostaining for cytokeratins as well as for other stem/progenitor cell marker.
5. *The Bowman capsule.* Mesenchymal stem/progenitor cells have been identified in recent years in the Bowman capsule of human glomeruli [32]. These mesenchymal progenitors have been shown to may originate from glomerular parietal epithelial cells through a process of epithelial-mesenchymal transition [33]. According with this hypothesis, glomerular parietal epithelial cells (PECs) might respond to renal injury by de-differentiating into embryonic phenotype or differentiating into podocytes [34]. At immunohistochemistry, PECs have been first reported to show reactivity for cluster of differentiation 24 (CD24) and prominin 1(CD133) [35]. In the preterm human kidney, PECs have been also shown to be reactive for neprilysin (CD109) [36]. Further studies showed that when activated, PECs acquire immunoreactivity for CD44, being this glycoprotein involved in cell adhesion and migration [37]. Recent data from our group confirm the presence of CD44+ cells even in preterm infants, suggesting that this peculiar stem cell niche also exists in the developing human kidney [18].
6. *Renal papilla.* The renal papilla was indicated as a possible niche for kidney stem cells more than 10 years ago [38]. Following studies evidenced the ability of these progenitors of the renal papilla to retain label from UB for a long time, differently from the major part of cells of the developing renal papilla [39]. Recent studies from our group evidenced a peculiar immunoreactivity for Tβ4in scattered cells of the renal papilla in preterm babies, confirming, at immunohistochemical level, the existence of a pool of renal progenitors in the human renal papilla [22].
  At histology, the renal papilla of preterm infants is characterized by a marked hyper-cellularity, as a compared to the papilla of adult kidneys. Many interstitial medullary cells show morphologic features similar to those observed in capsular stem/progenitors, confirming the hypothesis of the existence of a medullary stem cell niche in the developing kidney (Fig. 4). In recent years, CD133 has been indicated as a typical marker of stem/progenitor cells of the renal papilla, and their ability to be integrate into developing kidney tubules has been reported [40].

**Fig. 4:**
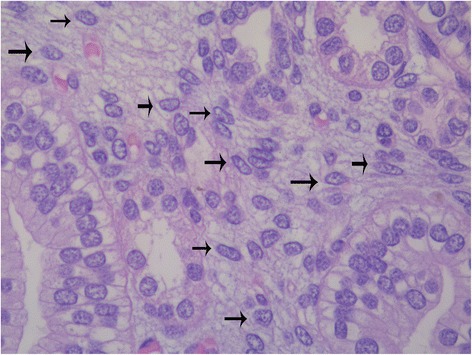
Interstitial stem/progenitor cells (*arrows*) in the renal medulla of a developing human kidney
7. *Hilum.* In recent years, the renal hilum has been indicated as a possible niche for a subset of renal stem cells. According with this hypothesis, stem/progenitor cells of the renal pelvis might originate the pacemaker cells that progressively populate the ureteral wall during development extending to the entire urinary tract [21]. Immunohistochemical studies have demonstrated that these pacemaker cells are characterized by the expression of the hyperpolarization-activated cation channel (HCN), that initiates urinaruy tract peristalsis [41]. Undifferentiated cells in the renal pelvis originate urinary pacemaker cells that propel urine from the kidney to the bladder. These cells express tyrosin-protein kinasi Kit (C-Kit), a tyrosine kinase also known as stem cell factor, whose expression is required for coordinated proximal to distal contraction of the ureter.

A question asked only recently regarding human kidnet development is “what factors determine cessation of nephrogenesis”? [42]. Regarding the stem cell pool located in the renal hilum, the question to be asked might be “is urinary pacemaker cell genesis blocked in preterms?” “Does prematurity determine cessation of urinary pacemaker cell differentiation?”.

### Which questions remain open to discussion regarding stem cells in the preterm kidney?

1. Are stem/progenitor cells of the multiple kidney niches derived by a common ancestor?
  All these data taken together, it emerges a new picture regarding stem/progenitor cell niches in the preterm kidney. There is not one stem cell niche. On the contrary, multiple stem cell niches may be identified in the fetal and preterm kidney. Stem/progenitors of the different niches differ significantly regarding morphology and immunohistochemistry. Each niche is characterized by the expression of specific molecules, which are related to the specific cell lineages that will originate by that specific stem cell niche. It is possible that all stem cells of all renal niches might derivate from a single common ancestor, the metanephric mesenchymal cells. At the present, a hierarchy of the multiple niches is not known.
2. Which is and where is located the “true” old metanephric mesenchymal progenitor? According with our knowledge, no data are available for the identification of remnant cell of the primitive metanephric mesenchyme. Capsular cells might represent it. This hypothesis should be confirmed by further studies at molecular level, able to define the hierarchy of the multiple pool of human renal stem cells.
3. Do other renal stem cell niches exist? In our opinion, the picture here described regarding the renal stem cell niche should be considered as incomplete, and esemplifies our ignorance regarding human nephrogenesis and, in particular, the complexity of the organization of renal progenitors in the human kidney. Further niches will be surely identified in next years. Classical morphology, associated to immune-histochemistry [43] and molecular biology [44] probably is not sufficient for reaching this goal. The triple I (interactive, intersectorial, interdisciplinary) approach is necessary for a better comprehension of nephrogenesis [45].
4. Which factors regulate exhaustion of stem/progenitor cells leading to cessation of nephrogenesis? The attention of perinatologists and nephrologists has been mainly focused, in recent years, on factors determining cessation of glomerulogenesis soon after birth, both in at term and in preterm neonates. Data here reported clearly indicate that the problem is more complex. The existence of multiple stem cell niches, apparently independent one from the next, lays stress on the opportunity of new questions: do single niches behave independently? May each single niche be silenced independently? Which factors determine the cessation of every stem cell niche? Which the consequences of a block of every stem cell niche and of each developmental compartment it represents?
5. Which is the role of medullary interstitial stem/progenitor cells in the perinatal remodeling of the medulla, allowing the neonatal kidney to become able to concentrate urine? The analysis of data regarding the progenitors of the renal interstitium evidences our ignorance regarding the progenitors of this important compartment, both in the cortex and in the renal medulla. Histology of the preterm kidney evidences the presence in the interstitium of the preterm kidney of a huge amount of undifferentiated cell types. Pivotal studies have recently shown the reactivity of these scarcely differentiated stromal cells for Tβ4 [22]. Further studies are needed in order to better characterize these interstitial cells and their role in kidney development, both in the cortex as well as in the renal papilla.

## Conclusions

A fascinating hypothesis is emerging in the field of perinatal nephrology. The possibility to act on the renal stem/progenitor cells in the perinatal period, helping them to complete their differentiation toward the multiple cell types that characterize the mature human kidney. This approach, that we previously defined as “physiological” regenerative medicine [13] underlines the diversity with previous regenerative approachs. Our project is based on the use of physiological tools, including the endogenous renal stem cells detectable in huge amounts in the kidney of all preterm infacnts. Maintaining stem cell activity after birth, particularly in neonates born preterm, might allow neonatologists to operate the primary prevention of chronic kidney disease later in life due to oligonephronia [23]. Data here reported induce to think that the problem of a physiological, endogenous regenerative medicine to be carried out in the newborn is probably more complex than previously thought. Until now, we considered nephrogenesis and stem/progenitors as a single target. Here we show that this is not the case, at least in the human kidney. The preterm kidney is characterized by the contemporary existence of multiple stem cell niches, all characterized by peculiar localization in peculiar renal compartments, by specific commitments toward typical cell types, and all identified by the expression of different molecular markers. As a consequence, our regenerative approach to be carried out in the preterm newborn should consider this complexity regarding the stem cell niches. Given the differences at morphological and immunohistochemical level of the multiple renal stem cells pools, we may speculate that the regenerative approach could be modulated according with the single stem cell pool we want to stimulate. Until now, the main target of our project was to implement the number of nephrons in preterms, starting a regenerative approach at birth, transforming their susceptibility into resistance to develop kidney disease later in life [23]. In previous studies, looking at human renal embryology with new eyes [46] we were able to hypothesize a previously unreported complexity in the organization of renal stem/progenitor cell niches. Recent studies have confirmed the complexity of the renal stem/progenitor cells niche, demonstrating that the contact between mesenchymal and epithelial progenitors gives rise to a complex exchange of morphogenetic information [47]. Recent immunohistochemical data and electron microscopic data clearly evidenced the strict association of the renal stem/progenitor cells niche with the renal capsule through microfibers originating from the basal lamina of each UB tip and extending toward the inner side of the renal capsule [48]. All these data taken together may represent the basis for a new original approach to the physiological regenerative medicine in preterms, low birth and very low birth weight infants.

## Abbreviations

UB: Ureteric bud
CKD: Chronic kidney disease
Mdm2: Mouse double minute 2
CD44: Cluster of differentiation 44
P53: Protein 53
Tβ4: Thymosin beta 4
H&E: Hematoxylin and eosin
Wnt1: Wingless-type MMTV integration site family, member 1
Bcl2: B-cell lymphoma2
MET: Mesenchymal-epithelial transition
Muc1: Mucin 1
FOXO3: Forkhead box 3
Pecs: Parietal epithelial cells
CD24: Cluster of differentiation 24
CD133: Prominin 1
CD10: Neprilysin
HCN: Hyperpolarization-activated cation channel
C-kit: Tyrosin-protein kinasi Kit
